# Innovative Field Applications of Probiotics, Prebiotics, and Medicinal Plant Products for Disease Control in Aquaculture

**DOI:** 10.1111/jfd.70227

**Published:** 2026-06-26

**Authors:** Mustafa Öz, Enes Üstüner, Sümmani Çifci, Suat Dikel, Emin İleri, Furkan Budak

**Affiliations:** ^1^ Department of Fisheries and Diseases, Faculty of Veterinary Medicine Aksaray University Aksaray Türkiye; ^2^ Department of Veterinary Parasitology, Faculty of Veterinary Medicine Aksaray University Aksaray Türkiye; ^3^ Department of Aquaculture, Faculty of Fisheries Cukurova University Adana Türkiye; ^4^ Department of Fisheries and Diseases, Graduate School of Health Sciences Aksaray University Aksaray Türkiye

**Keywords:** disease control, fish health, medicinal plants, prebiotics, probiotics, sustainable aquaculture

## Abstract

Disease outbreaks and the associated reliance on antibiotics pose major constraints to the sustainability of modern aquaculture. As regulatory pressures increase and consumer demand shifts toward residue‐free production, diverse biological interventions are gaining prominence as viable alternatives to chemotherapeutics. These include microbiome‐modulating agents (probiotics, classical prebiotics, and synbiotics), alongside a distinctly separate category of functional additives: phytogenics (medicinal plant derivatives), which are valued for their direct bioactive and immunomodulatory properties. This review synthesizes current laboratory and field‐based evidence regarding the efficacy, mechanisms, and practical challenges of these functional additives. While in vitro and controlled studies demonstrate clear benefits in immune modulation, competitive exclusion, and gut health, real‐world application is frequently hindered by environmental inconsistencies and formulation instability. We critically evaluate the impact of system‐specific variables and draw several specific mechanistic inferences: First, the over‐reliance on static in vitro assays fundamentally fails to predict in vivo colonization under multifactorial field stress (e.g., thermal and pH fluctuations). Second, thermal degradation during industrial feed extrusion is a primary driver of batch‐to‐batch inconsistency, rendering advanced microencapsulation and post‐coating techniques practically mandatory for viable delivery. Third, the efficacy of functional additives is strictly governed by the culture matrix; while the chemical stability of Recirculating Aquaculture Systems (RAS) yields predictable outcomes, open ponds face severe abiotic fluctuations, and Biofloc Technology (BFT) requires precise Carbon‐to‐Nitrogen (C:N) stoichiometry to facilitate heterotrophic assimilation. To overcome these limitations, we propose a strategic shift toward next‐generation interventions specifically thermal‐stable postbiotics, precision phage therapy for niche‐clearing, and rigorous multi‐omics technologies for molecular validation, replacing purely phenotypic observations. By integrating these specific innovations within a precision digital health framework, this work provides a comprehensive roadmap for standardizing bio‐based disease control for sustainable and reproducible aquaculture production.

## 1 Introduction

Aquaculture production continues to expand rapidly and has become a critical component of global food security (FAO 2024). However, the intensification of production systems characterized by high stocking densities, fluctuating environmental conditions, and operational stressors has considerably increased the susceptibility of fish to pathogenic infections (Das et al. 2017). Stress‐induced immunosuppression creates favourable conditions for bacterial, viral, and parasitic diseases. These conditions result in recurrent outbreaks that threaten productivity, animal welfare, and the long‐term sustainability of the sector (Ciji and Akhtar 2021; Dawood et al. 2018).

For decades, antibiotics have been the primary means of disease control in aquaculture. Yet projections indicate that antibiotic use in the industry may increase by 8% and reach approximately 108 metric tons by 2030 (Mulchandani et al. 2023). Growing evidence linking antibiotic use to antimicrobial resistance, environmental contamination, and residue‐related public health risks has intensified calls for safer and more sustainable alternatives (Qu et al. 2025). As a result, biological strategies capable of enhancing fish health and reducing reliance on antibiotics have gained significant momentum. Among these strategies, probiotics, prebiotics, synbiotics, and medicinal plant products have emerged as promising tools.

Probiotics are non‐pathogenic microorganisms that provide functional benefits by supporting host immunity, stabilizing gut microbiota, and suppressing pathogens through competitive exclusion and the production of antimicrobial metabolites (Banerjee and Ray 2017; Dawood et al. 2018; Madhulika et al. 2025). Prebiotics such as oligosaccharides serve as selective substrates for beneficial microbes, which enhance gut integrity and digestive efficiency (Davani‐Davari et al. 2019; Wee et al. 2024). Meanwhile, phytogenic compounds and medicinal plant extracts offer antioxidant, anti‐inflammatory, antimicrobial, and immunostimulatory effects that contribute to improved disease resistance and physiological resilience (Zhu 2020; Wei et al. 2022). Together, these natural additives represent important components of a sustainable and antibiotic‐free health management framework for aquaculture.

Despite robust laboratory evidence, real‐world applications of these functional additives frequently yield inconsistent results. A major methodological weakness in the current literature is the over‐reliance on simplistic in vitro assays to predict in vivo efficacy. These static models fail to account for the complex molecular interactions within the host gut and the highly variable environmental conditions (e.g., fluctuating temperature, salinity, and pH) of commercial systems (Calcagnile et al. 2024; Rahayu et al. 2024). These static models fail to account for the complex molecular interactions within the host gut and the highly variable environmental conditions (e.g., fluctuating temperature, salinity, and pH) of commercial systems (Calcagnile et al. 2024; Rahayu et al. 2024). Recent primary research strictly confirms this discrepancy. For example, specific probiotic strains exhibiting potent in vitro antagonism against *Vibrio* spp. and *Saprolegnia parasitica* completely failed to confer survival benefits during in vivo challenge trials in shrimp and rainbow trout, respectively (Fregeneda‐Grandes et al. 2023; Thompson et al. 2022). Furthermore, environmental constraints such as elevated salinity have been proven to inhibit the in vivo expression of targeted antimicrobial metabolites even when the probiotic strain remains viable (Mirbakhsh et al. 2022). Furthermore, many studies merely report phenotypic improvements, such as enhanced feed conversion ratios (FCR) or survival rates, without elucidating the underlying molecular mechanisms such as specific immunomodulatory pathways, competitive exclusion dynamics, or metabolic cross‐feeding. This heavily descriptive approach in the literature masks the fundamental reasons why certain biological interventions fail under multifactorial field stress. Therefore, rather than providing a descriptive summary of successful trials, this review critically analyses the *how* and *why* behind the field performance of probiotics, prebiotics, and medicinal plants. We critically evaluate the mechanistic pathways governing host‐microbiome‐pathogen interactions and pinpoint the specific methodological flaws and environmental variables responsible for farm‐to‐farm variability. By establishing this critical analytical framework, we strategically position emerging fields such as microencapsulation technologies and precision digital health monitoring not as separate, broad topics, but as targeted, mechanistic solutions required to overcome product instability and ensure reproducible disease control in sustainable aquaculture.

## 2 From Laboratory Promise to Farm Practice

### 2.1 Probiotics as Living Defenders

The survival rate of probiotics becomes lower when they experience high pressure and temperature conditions which occur during pelleting and extrusion operations until they receive protective formulations. The researchers achieved two main research goals through their experimental design which protected therapeutic cell numbers and enhanced methods for gut delivery. The researchers reached their targets by using biopolymer encapsulation and protecting cells through low‐temperature handling and spray‐drying with protective matrices and optimized extrusion parameters (Gede et al. 2024; Vijayaram et al. 2024). However, this physical constraint does not apply universally across all forms of aquaculture, as the optimal route of administration is strictly dictated by the targeted species and the specific culture system design. For instance, in intensive Biofloc Technology (BFT) and Recirculating Aquaculture Systems (RAS), functional microbes are frequently applied as direct water additives rather than through feed. In closed biofloc systems utilized for tropical shrimp ( *Penaeus vannamei* ) aquaculture, specific probiotic bacteria and nitrifying consortia (e.g., *Nitrosomonas* and *Nitrobacter*) are inserted directly into the water system to facilitate floc formation and maintain water quality, entirely bypassing the harsh conditions of feed manufacturing. Therefore, resolving viability issues requires researchers to match the administration strategy whether employing biopolymer encapsulation for direct gut delivery in pond‐reared finfish or utilizing water‐borne inoculation for environmental bioremediation in shrimp biofloc systems to the specific technological constraints of the farm (Papadopoulos et al. 2024). The probiotic strains *Bacillus* spp. (The list includes *B. subtilis* as well as *Lactobacillus* and *Enterococcus* species from lactic acid bacteria and Pseudoalteromonas spp. which function as marine antagonists). The research showed that *Lactobacillus* strains successfully prevented *Aeromonas* and *Vibrio* and *Streptococcus* from obtaining resources according to results from laboratory tests and animal‐based research (Garcés et al. 2020; Suria et al. 2020; Waiyamitra et al. 2020). The high number of animals in the area together with better aquaculture practices have altered how nutrients and disease‐causing pathogens spread through the environment. The development of probiotics and their immune system advantages depends on these elements which force scientists to pick certain bacterial strains and measure exact amounts for various uses (Hasan and Banerjee 2020; Zhang et al. 2021). The use of protected feed formulations which contain micro‐encapsulation and pectin‐based pellets together with environmental improvements that boost competitive exclusion in ponds enables reliable dosing for achieving successful results (Gede et al. 2024; Said et al. 2022; Vijayaram et al. 2024). Research studies show that these bacterial strains function as successful treatments for particular infections when deployed in different outdoor environments. Table 1 provides a complete overview of probiotic uses which includes particular treatment amounts and survival rates for various fish species.

**TABLE 1:** Summary of recent field applications of probiotic strains in aquaculture, detailing dosage, administration routes, and observed disease control outcomes (2020–2025).

Aquatic species	Probiotic Strain(s) (e.g., Bacillus, Lactobacillus)	Dosage & administration route	Target pathogen(s)	Key results (survival rate, immune response)	Mechanisms of action	Limitations/field challenges	Citations
Largemouth bass ( *Micropterus salmoides* )	*Pediococcus acidilactici* B49	8‐week oral feeding	*Aeromonas hydrophila*	↑ Survival, ↑ lysozyme, ↑ IL‐8, ↓ TGF‐β, ↑ IgM, ↑ phagocytosis	Lactic acid production, local pH reduction, PRR activation	High thermal sensitivity during standard extrusion processing	Soto‐Dávila et al. (2024)
Largemouth bass ( *Micropterus salmoides* )	*Enterococcus lactis* A1 (GABA‐producing)	8‐week oral feeding	*Aeromonas hydrophila*	↑ Survival, ↑ SOD/catalase, ↑ trypsin/lipase, ↑ villus height, ↑ immune response	GABA synthesis, competitive exclusion on intestinal mucosa	Low viability without encapsulation; gastric acid susceptibility	Hou et al. (2025)
Large yellow croaker ( *Larimichthys crocea* )	*Lactobacillus sakei* YLD10	56‐day oral feeding	*Pseudomonas plecoglossicida*	↑ Survival, ↑ digestive enzymes, ↑ antioxidant capacity, ↑ non‐specific immunity, ↓ tissue burden	SCFA production fueling enterocytes, gut barrier reinforcement	Fails to colonize dynamically changing marine salinities	Saba et al. (2024)
Nile tilapia ( *Oreochromis niloticus* )	*Bacillus subtilis* , *Bacillus amyloliquefaciens* , *Bacillus* sp	Feed‐based (various durations)	*Streptococcus, Aeromonas*	↑ Survival, ↑ immune modulation, ↑ growth, ↓ pathogen load	Secretion of antimicrobial lipopeptides (bacteriocins), TLR signalling	Germination failure if gut pH/temperature is suboptimal	Koga et al. (2022), Qiu et al. (2023), Raheem et al. (2021), Romanova et al. (2022)
White shrimp ( *Litopenaeus vannamei* )	*Bacillus licheniformis* , *B. amyloliquefaciens* , *B. subtilis* , *Pseudomonas* sp. (consortium SFSK4)	Water additive (10^7^ CFU/mL)	*Vibrio* spp.	↑ Immunity (hemocyte count, phagocytosis), ↑ phenoloxidase, ↓ mortality (> 50%), no toxicity	Quorum quenching, competitive exclusion of *Vibrio* in water column	Washed out rapidly in high‐exchange water systems	Ringø et al. (2022)
Shrimp (various species)	*Bacillus, Lactobacillus, Enterococcus, Pseudomonas, Roseobacter*, yeast (e.g., *Saccharomyces cerevisiae* )	Feed or water additive (varied)	*Vibrio* spp., AHPND, WSSV	↑ Survival, ↑ immune response, ↑ gut health, ↓ pathogen prevalence	Synergistic immune priming, MAMP‐mediated GALT activation	Batch‐to‐batch inconsistency; inter‐strain competition	Fachri et al. (2024), Fernandes et al. (2021), Gaffar et al. (2023), Golder et al. (2022), Han et al. (2024), Khanjani et al. (2024), Madhulika et al. (2025), Mohammed, Ahmed, et al. (2025), Mohammed, Kovács, and Pál (2025), Paritova et al. (2024), Pérez‐Jiménez et al. (2024), Wu et al. (2021)
Finfish (various species)	*Bacillus, Lactobacillus, Enterococcus, Carnobacterium, Phaeobacter, Roseobacter*	Feed additive, water additive	*Vibrio*, *Aeromonas*, *Tenacibaculum*, *Streptococcus*	↑ Survival, ↑ immune response, ↑ growth, ↓ pathogen colonization	Native mucosal adhesion, suppression of pathogenic virulence genes	High host specificity limits broad‐spectrum commercialization	Ahmmed et al. (2023), Ehsannia et al. (2022), Koga et al. (2022), Kuebutornye et al. (2020), Langlois et al. (2021), Singh et al. (2024), Wang et al. (2025), Yang et al. (2022)
Shellfish (shrimp, prawn, crab, oyster, abalone)	*Bacillus, Lactobacillus, Pseudoalteromonas, Enterobacter*, yeast	Feed or water additive	*Vibrio* spp., WSSV, YHD	↑ Survival, ↑ immune modulation, ↑ growth, ↓ viral/bacterial disease	ProPO cascade activation, competitive spatial exclusion.	Variable viability in highly diverse aquatic environments.	Ahmmed et al. (2023), Kuebutornye et al. (2020), Mohammed, Ahmed, et al. (2025), Mohammed, Kovács, and Pál (2025), Wu et al. (2021)
Mixed algal microbiomes (*Tetraselmis suecica*, *Isochrysis galbana* )	*Alteromonadaceae, Halomonadaceae, Rhodobacteraceae, Flavobacteriaceae, Sulfitobacter, Vreelandella*	In vitro co‐culture	*Vibrio anguillarum*	↑ Pathogen inhibition (synergistic effect in co‐culture)	Microalgae‐bacteria synergism, nutrient and spatial competition.	Difficult to maintain stable co‐cultures at commercial‐scale	Tachibana et al. (2020)
Giant freshwater prawn ( *Macrobrachium rosenbergii* )	*Bacillus, Lactobacillus, Enterococcus*	Feed additive, water additive	White tail disease, WSSV, bacteria	↑ Immunity, ↑ survival, ↓ disease incidence	Modulation of innate cellular immunity and hemocyte proliferation.	Strain degradation during prolonged feed storage.	Contente et al. (2023)

### 2.2 Prebiotics as Microbial Architects

Prebiotic oligosaccharides (Mannan‐oligosaccharides (MOS), Fructo‐oligosaccharides (FOS), Xylo‐oligosaccharides (XOS), Galacto‐oligosaccharides (GOS), Gluco‐oligosaccharides (GlcOS)) exist as undigestible carbohydrates which support particular gut bacteria while their chemical structure remains unaltered during production and standard animal feed operations. The structural chains of these materials become unstable when exposed to extreme steam or shear forces or high moisture levels unless they receive protection or application after the extrusion process (Banafsha and Reddy 2025; Zeng et al. 2023). Research studies conducted in controlled trials demonstrate that while standalone prebiotics at low dietary inclusion levels (low g/kg) effectively improve gut health by selectively stimulating the endogenous (native) microbiota, superior and more predictable outcomes are frequently achieved through synbiotic applications where the prebiotic is intentionally combined with a co‐administered exogenous probiotic strain to ensure directed fermentation (Banafsha and Reddy 2025). Water bodies with high ammonia or nitrite concentrations and low dissolved oxygen levels cause changes in microbiota populations which result in decreased prebiotic fermentation and impaired immune system regulation. The RAS and biofloc systems operate as buffers which stabilize water chemistry and generate more stable responses (Flo et al. 2024; Maduka et al. 2022; Rizki et al. 2024). The deployment of protected or post‐coated feed inclusion and biofloc systems and synbiotic product formulation needs site‐level pilot trials to confirm dosage and delivery methods before moving to extensive commercial use (Banafsha and Reddy 2025; Flo et al. 2024; Zeng et al. 2023). The implementation of probiotics and prebiotics in aquaculture requires understanding their synergistic benefits, from protective formulations to system optimization, as illustrated in Figure 1. While physiological benefits are well‐documented, the impact of these prebiotics extends to environmental parameters. Table 2 provides a detailed comparison of prebiotic types, their inclusion levels, and their dual impact on host gut health and system water quality.

**FIGURE 1:**
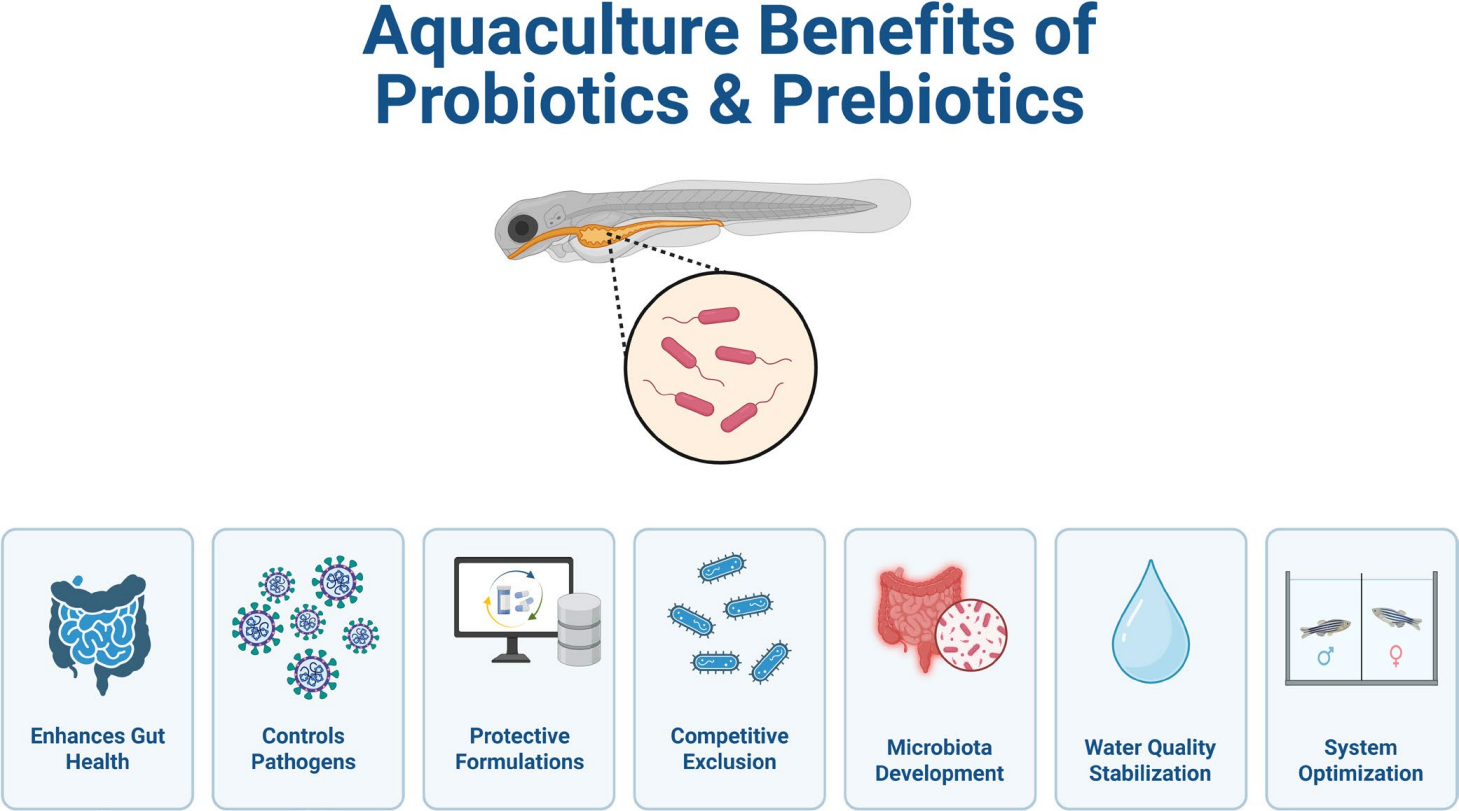
Comprehensive benefits of probiotics and prebiotics in aquaculture systems. The integrated approach demonstrates seven key advantages: (1) Enhances Gut Health through selective stimulation of beneficial intestinal microbiota; (2) Controls Pathogens via competitive exclusion against Aeromonas, Vibrio, and Streptococcus species; (3) Protective Formulations utilizing biopolymer encapsulation and optimized processing parameters; (4) Competitive Exclusion where beneficial bacteria outcompete pathogenic microorganisms; (5) Microbiota Development through prebiotic oligosaccharides (MOS, FOS, XOS, GOS, GlcOS); (6) Water Quality Stabilization using RAS and biofloc systems as buffers; (7) System Optimization through integrated delivery methods combining protected feed formulations with environmental enhancements.

**TABLE 2:** Overview of prebiotic interventions (MOS, FOS, XOS) in aquaculture systems: Effects on gut microbiota diversity, water quality parameters, and immune modulation.

Target species	Prebiotic formulation (standalone vs. synbiotic) & level	Effects on gut microbiota diversity	Impact on water quality parameters	Disease resistance outcomes	Mechanisms of action	Limitations/field challenges	Citations
*Sparus aurata* , *Dicentrarchus labrax* (sea bream, sea bass)	Standalone Prebiotics (MOS, FOS, Inulin: 0.2%–1% of diet)	Increased beneficial bacteria (e.g., Lactobacillus, Bifidobacterium); improved gut morphology	Not directly measured; prebiotics considered eco‐friendly	Enhanced innate immunity (phagocytosis, lysozyme, complement); improved survival against pathogens	Pathogen agglutination via mannose receptors; SCFA generation	Thermal degradation during pelleting shear stress.	Carbone and Faggio (2016), Guerreiro et al. (2018), Ringø et al. (2010), Song et al. (2014)
Carp species (Cyprinidae)	Mixed (Standalone MOS, FOS: 0.2%–1%; often combined with *Bacillus* as Synbiotics)	Modulation of gut microbiota; increased lactic acid bacteria; reduced pathogens	Noted reduction in ammonia/nitrite in some studies	Improved immune response (lysozyme, phagocytosis); higher survival after bacterial challenge	Selective substrate for endogenous lactic acid bacteria	Efficacy is strictly dependent on the host's baseline microbiome	Amenyogbe et al. (2024), Dawood and Koshio (2016), Mohan et al. (2019), Ringø et al. (2010), Wee et al. (2024)
Tilapia ( *Oreochromis niloticus* )	Standalone Prebiotics (MOS 5 g/kg, FOS, β‐glucan, XOS)	Altered gut microbial composition; increased beneficial bacteria; improved villus length	Some studies show reduced ammonia, improved water parameters	Enhanced disease resistance (lower mortality after *Vibrio*/*Aeromonas* challenge); improved immune gene expression	Upregulation of tight junction proteins; local mucosal priming	Overdosing (> 2%) causes mucosal exhaustion and FCR deterioration	Elsabagh et al. (2018), Wang et al. (2022), Xu et al. (2022), Zhu et al. (2023)
Hybrid grouper (*Epinephelus* spp.)	Standalone Prebiotics (MOS 0.2%, FOS 0.2%, β‐glucan 0.1%)	MOS and XOS increased gut microbiota diversity and villus length	Not directly measured	MOS and β‐glucan groups had higher survival after pathogen challenge; improved antioxidant and immune status	PRR binding (e.g., Dectin‐1 for β‐glucan), activation of systemic phagocytes	High production cost of purified oligosaccharides	Zhu et al. (2023)
Pacific white shrimp ( *Litopenaeus vannamei* )	Mixed (Standalone Inulin/MOS 2.5–10 mg/g; sometimes paired with *Lactobacillus*)	Combined inulin+MOS increased beneficial gut bacteria and immune gene expression	Improved water quality (lower ammonia, better clarity) in some studies	Lower mortality after WSSV and Vibrio challenge; enhanced immune gene expression	Synergistic activation of the proPO cascade and hemocytes	Unassimilated residues can trigger opportunistic pathogen blooms.	Chang et al. (2025), Li et al. (2018), Noman et al. (2024)
Atlantic salmon ( *Salmo salar* )	Standalone Prebiotic (Alginate oligosaccharide 0.5%–2.5%)	Low‐level AlgOS increased beneficial butyrate‐producing bacteria; altered microbiota composition	Not directly measured	Improved gut health; potential for increased disease resistance	Shifts metabolic pathways toward enhanced butyrate production	Difficult to standardize oligosaccharide chain lengths at industrial scale	Gupta et al. (2019)
Caspian trout (*Salmo trutta caspius*)	Standalone Prebiotics (MOS 4 mg/g, β‐glucan 3 mg/g)	Enhanced intestinal microbial load of beneficial bacteria	Not directly measured	Enhanced humoral innate immunity; upregulation of immune‐related genes	Enhancement of phagocytic cell activity and systemic leukocyte trafficking	Dose‐dependent efficacy variations under thermal stress	Jami et al. (2019)
General (multiple species: catfish, sturgeon, etc.)	Mixed (Both Standalone Prebiotics and Synbiotic combinations)	Selective stimulation of beneficial bacteria; increased microbial diversity	Some studies report improved water quality (lower ammonia/nitrite)	Improved growth, immune response, and survival across species	Cross‐feeding networks among commensal gut microbes.	Unpredictable interactions with fluctuating pond water chemistry	Amillano‐Cisneros et al. (2023), Ganguly et al. (2013), Hoseinifar et al. (2016), Mohapatra et al. (2013)

### 2.3 Medicinal Plants as Phytogenic Protectors

To establish conceptual clarity, it is crucial to explicitly differentiate medicinal plant derivatives (often termed phytogenics or phytobiotics) from classical prebiotics, as they are not a subcategory of the latter. While prebiotics are primarily non‐digestible carbohydrates that serve merely as selectively fermented metabolic substrates to feed and promote beneficial gut bacteria, medicinal plant extracts operate through fundamentally different molecular pathways. They are rich in bioactive secondary metabolites such as essential oils, phenolics, flavonoids, and alkaloids which exert direct pharmacological effects. Rather than feeding the microbiota, these phytogenic compounds provide direct bactericidal activity, disrupt pathogen quorum sensing, and directly activate host immune receptors. Following this distinction, numerous botanical extracts have been proven to exhibit these specific, direct beneficial properties in aquaculture and fishery products (Esen et al. 2025; Öz, Inanan, et al. 2024; Öz, Üstüner, and Bölükbaş 2024; Öz, Üstüner, Çifci, et al. 2025; Öz, Üstüner, Jumayeva, and Dikel 2025; Tok et al. 2025). Local plants provide supply chain and cultural advantages to the market but imported concentrates must undergo complete authentication procedures because they present potential risks to authenticity and safety (Ara et al. 2020). The feed industry uses aqueous or methanolic extraction methods as part of its standard workflow which includes concentration/solvent removal and phytochemical profiling through HPTLC and DNA barcoding for species verification (Ara et al. 2020; Ojha et al. 2020; Terzi et al. 2023). Standardized supplements have proven effective for weight gain and feed conversion and disease resistance in different species yet their effectiveness depends on species type and dosage and processing methods and needs on‐farm testing for validation and sustainable supply systems (Ahmad et al. 2024; Latif et al. 2020; Nuwagira et al. 2022). The bioactive potential of these phytogenics varies significantly based on the plant part and extraction solvent used. Table 3 synthesizes recent findings on medicinal plant applications, highlighting the correlation between extraction methods and disease resistance outcomes.

**TABLE 3:** Efficacy of medicinal plant extracts and phytogenic additives in aquaculture: Plant parts used, extraction methods, and reported immunostimulatory properties against common pathogens.

Plant species & part used	Extraction method (aqueous/methanolic/other)	Aquatic species	Dietary inclusion level	Specific immune/antioxidant benefits	Resistance against pathogens/outcomes	Mechanisms of action	Limitations/field challenges	Citations
*Azadirachta indica* (Neem, leaves), *Withania somnifera* (roots), *Allium sativum* (Garlic, bulbs), *Zingiber officinale* (Ginger, rhizome), *Ocimum sanctum* (leaves), *Tinospora cordifolia* (stem), *Aloe barbadensis* (leaves)	Aqueous, methanolic, ethanolic, essential oil	Various fish (e.g., carp, tilapia, catfish, pangasius)	0.5%–5% of diet, or as extracts in water	Immunostimulant, antioxidant, anti‐stress, growth‐promoting, increased lysozyme, phagocytosis, cytokine expression	Enhanced resistance to *Aeromonas hydrophila* , *Vibrio* spp., *Edwardsiella*, *Saprolegnia*, reduced mortality	Cell wall disruption of Gram‐negative bacteria (phenolics); ROS scavenging	High volatility during processing; seasonal phytochemical variation	Ahmadi et al. (2012), Dawood et al. (2022), Ghosh et al. (2025), Harikrishnan et al. (2011), Munaeni et al. (2021), Rashidian et al. (2021), Salomón et al. (2020), Semwal et al. (2023), Shadmand et al. (2025), Tzortzatos et al. (2024), Yang et al. (2024), Zhu (2020)
*Curcuma longa* (Turmeric, rhizome), *Phyllanthus amarus* (whole plant), *Tinospora cordifolia* (stem)	Methanolic, ethanolic, aqueous	Shrimp ( *Litopenaeus vannamei* ), carp, tilapia	0.5%–5% of diet, 250 mg/kg feed	Enhanced total hemocyte count, phagocytosis, lysozyme, glutathione peroxidase, reduced oxidative stress	Reduced *Vibrio parahaemolyticus* , *Aeromonas*, White Faeces Syndrome, improved survival	Inhibition of quorum sensing (quorum quenching); GALT activation	Poor aqueous solubility (curcumin); rapid systemic clearance	Afonso et al. (2021), Arena et al. (2022), Ghosh et al. (2025), Rashidian et al. (2021), Tarricone et al. (2023)
*Murraya koenigii* (Curry leaf, leaves), *Tagetes erecta* (Marigold, leaves)	Methanolic, ethanolic	*Labeo rohita* (rohu)	1%–2% of diet, combined extracts	Increased haemoglobin, leukocyte count, immunoglobulin, stable liver enzymes, enhanced survival	Resistance to *Saprolegnia parasitica* , higher survival and lower lesion severity	Stabilization of hepatocyte membranes; direct fungicidal activity	Extraction solvents can leave toxic residues if not purged	Guo et al. (2023)
*Artemisia herba‐alba* (whole plant), *Lonicera japonica* (flowers), *Lilium candidum* (bulbs)	Methanolic, aqueous	Shrimp ( *L. vannamei* )	250 mg/kg feed	Improved antioxidant enzymes (catalase, SOD), lysozyme, total protein, immune cell counts	Reduced *Vibrio parahaemolyticus* mortality, milder disease symptoms	Upregulation of antioxidant gene clusters; enhancement of hemocytes	Bioactive compounds degrade rapidly under UV and oxygen exposure.	Tarricone et al. (2023)
*Psidium guajava* (Guava, leaves), *Phyllanthus acidus* (Star gooseberry, leaves)	Methanolic, ethanolic	Nile tilapia ( *Oreochromis niloticus* )	10 g/kg feed, 0.2%–1% of diet	Enhanced antimicrobial activity, improved immune‐haematological parameters, altered gut microbiota	Increased resistance to *Aeromonas hydrophila* , higher survival	Direct bactericidal activity via membrane permeabilization	Tannin‐induced feed palatability issues at high doses	Ahmadi et al. (2012), Munaeni et al. (2021), Tarricone et al. (2023)
*Avicennia officinalis* (Mangrove, leaves)	Ethyl acetate, aqueous	Freshwater fish (various)	2%–8% of feed	Antibacterial, improved immunity, decreased mortality	Effective against *Aeromonas hydrophila* , *A. liquefaciens* , *Pseudomonas fluorescens*	Disruption of bacterial biofilms on mucosal surfaces	Inconsistent bioactive concentrations across geographical regions	Andriani and Aisyah (2025)
*Ficus carica* (fig, polysaccharides), Radix isatidis, Schisandra chinensis	Polysaccharide extraction	Crucian carp	Not specified	Enhanced phagocytosis, lysozyme, complement, SOD, total protein	Reduced *Aeromonas hydrophila* mortality, improved survival	Polysaccharide‐mediated PRR activation on macrophages	Complex and expensive extraction processes scale poorly	Afonso et al. (2021)
Mixed herbal blends (e.g., turmeric, garlic, *aloe vera* )	Aqueous, methanolic, essential oil	*Pangasius hypophthalmus* , carp, tilapia	5% of diet, variable	Improved growth, survival, immune indices, antioxidant status	Broad resistance to bacterial and fungal pathogens, reduced FCR	Synergistic multi‐receptor immune activation; gut microbiota alteration	Antagonistic interactions between unpurified crude extracts	Ghosh et al. (2025), Munaeni et al. (2021), Rashidian et al. (2021)
Essential oils (e.g., from Lamiaceae, Asteraceae, citrus)	Essential oil extraction	European seabass, carp, tilapia	200–1000 ppm in feed	Lower stress (cortisol), increased IgM, upregulated immune genes, antioxidant protection	Enhanced vaccine efficacy, resistance to *Vibrio anguillarum*	HPI‐axis (stress) modulation; pathogen membrane permeabilization	Extremely volatile; requires advanced microencapsulation for shelf life	Thangadurai and Puvaneswari (2022), Yousefi et al. (2025)

### 2.4 Bioremediation Mechanisms: Nitrification and Water Quality Control

The water quality management in aquaculture systems depends on probiotics and prebiotics because these substances influence biogeochemical cycles through their control of nitrogen cycle operations. The operational stability of closed systems, particularly Recirculating Aquaculture Systems (RAS) and Biofloc Technology (BFT), relies heavily on complex microbial community dynamics to mitigate the continuous accumulation of toxic nitrogenous wastes. In RAS, bioremediation is driven by autotrophic nitrification within biofilters. Rather than simply converting ammonia, the inoculation of specific Ammonia‐Oxidizing (AOB) and Nitrite‐Oxidizing (NOB) bacterial consortia fundamentally accelerates biofilm maturation kinetics. A critical mechanistic challenge in RAS is managing the organic carbon load; excessive carbon allows fast‐growing heterotrophic bacteria to spatially and nutritionally outcompete the slower‐growing autotrophic nitrifiers for oxygen and attachment sites within the biofilm matrix, thereby destabilizing the biofilter (Almeida et al. 2015). Conversely, Biofloc Technology operates on a distinctly different ecological principle: the stoichiometric manipulation of the Carbon‐to‐Nitrogen (C:N) ratio. By supplementing systems with prebiotic carbohydrate sources (e.g., molasses or precise oligosaccharide profiles) to maintain a C:N ratio above 15:1, the metabolic pathway is forcibly shifted from autotrophic nitrification to heterotrophic assimilation. This forces Bacillus spp. and other heterotrophs to actively sequester inorganic nitrogen directly from the water column to synthesize cellular protein. Furthermore, this process is strictly dependent on the microbial secretion of Extracellular Polymeric Substances (EPS). The EPS matrix acts as a functional architectural scaffold, aggregating suspended organic matter, algae, and diverse bacterial taxa into structurally robust macro‐flocs. This intricate microenvironment not only facilitates niche partitioning allowing localized micro‐aerobic and anaerobic zones to coexist but also serves as highly bioavailable, continuously produced supplemental nutrition for the host. Recent microbial analyses indicate that precisely engineered carbohydrate dosing, combined with microalgae, significantly optimizes this EPS architecture and bacterial community assembly, enhancing systemic resilience in *Litopenaeus vannamei* cultivation (Raza et al. 2025).

The system benefits from probiotic strains which operate in pond sediment areas with low oxygen levels to perform denitrification which transforms nitrate into nitrogen gas that completely eliminates system nitrogen excess. The enzymatic system functions as an essential mechanism which stops nitrates from building up while it helps static ponds to preserve their water stability during prolonged operations (Hucheng et al. 2020). The three pathways of nitrification, heterotrophic assimilation, and denitrification enable biological additives to convert waste management into an ecosystem service.

### 2.5 Laboratory and Field Performance Gaps

The transition of laboratory achievements into commercial success becomes difficult because farms function under different ecological conditions, demographic factors, and operational parameters which differ substantially from laboratory environments. The laboratory environment provides stable chemical conditions together with uniform population ages and precise medication delivery, but commercial aquaculture operations must handle changing dissolved oxygen levels and unpredictable ammonia concentrations, sediment accumulation, and various natural microbial communities. The survival and colonization of probiotics within the host depends on particular environmental elements which (Nguyen et al. 2022; Ringø et al. 2020; Vijayaram et al. 2024).

A critical assessment of recent literature reveals severe methodological flaws in how functional additives are screened and evaluated, which explains the high failure rate in actual field conditions. The fundamental weakness lies in selecting probiotic strains based solely on in vitro agar plate inhibition zones. This deeply flawed methodology falsely equates simple, static chemical antagonism with complex in vivo efficacy. These selected strains frequently fail in the field because they lack the molecular mechanisms required to adhere to the host mucosal epithelium or outcompete the established, highly resilient native microbiota present in complex matrices like biofloc or pond sediments (Calcagnile et al. 2024; Rahayu et al. 2024). Furthermore, many studies disregard the catastrophic impact of industrial scale‐up. Extrusion processing involves high shear forces and elevated temperatures that fundamentally denature phytogenic active compounds and decimate vegetative bacterial viability, rendering standard formulations essentially inert before they even reach the water. The failure to account for these processing losses, coupled with a lack of standardized multi‐omics validation, leads to unpredictable batch‐to‐batch variations that plague current commercial applications (Choi et al. 2022; Hernandez‐Patlan et al. 2022; Martínez‐Ángeles et al. 2023).

The survival of allochthonous probiotics in outdoor environments faces two main biological obstacles because they require particular host strains and must fight against the dominant native microbial community (Delgado et al. 2020; Du et al. 2021; Kang et al. 2022). The effectiveness of probiotics depends on seasonal and climatic changes which impact temperature levels, oxygen concentrations, and nutrient availability. Research needs to establish selection criteria which unite laboratory evaluation with stress resistance testing to create new farming methods which handle both commercial‐scale cultivation and periodic changes in crop availability (Nguyen et al. 2022; Ringø et al. 2020; Vijayaram et al. 2024).

## 3 Field Evidence and Real‐World Applications

### 3.1 Comparative Analysis of Field Trials

Before evaluating specific field outcomes, it is critical to address the commercial reality of biological additive adoption across different aquaculture clades. Current market surveys and bibliometric data reveal a massive discrepancy between the rapidly growing volume of academic research and actual field‐level implementation (Mohammed, Ahmed, et al. 2025; Mohammed, Kovács, and Pál 2025). While hundreds of experimental studies continuously highlight promising candidate strains, the translation of these laboratory successes into standardized, commercially available products remains severely limited (Amenyogbe 2023; Rahayu et al. 2024). Recent field surveys validate this gap; for example, a study assessing commercial fish farmers demonstrated that while an overwhelming majority (over 92.1%) held highly favourable perceptions of probiotics, the actual real‐world application remained strikingly low (Akter et al. 2026). In specific freshwater finfish sectors, the documented usage of in‐pond probiotics as alternatives to conventional chemicals is reported to be only 12% among producers (Rasul et al. 2025). This stark contrast between high theoretical acceptance and low practical adoption powerfully supports the core thesis of this review: severe environmental inconsistencies and a lack of reliable, field‐ready delivery protocols actively deter widespread commercial integration (Amenyogbe 2023; Rahayu et al. 2024).

The results of field tests show that commercial products made from single‐strain and multi‐strain spore‐forming *Bacillus* species effectively decrease disease occurrence. The research shows that *Latilactobacillus*/*Lactobacillus* and *Pseudoalteromonas* and *Carnobacterium marine* antagonists work best in specific situations (Benedetti et al. 2024; Cathers et al. 2022). While both operate as closed environments, the ecological roles of biological additives in Recirculating Aquaculture Systems (RAS) and Biofloc Technology (BFT) must be strictly differentiated. In RAS, the highly controlled water chemistry primarily acts as a stable abiotic buffer, which minimizes stress and allows prebiotics and probiotics to create steady, predictable microbiota shifts within the host. Conversely, in BFT, functional microbes are fundamentally indispensable to the system's architecture; heterotrophic bacteria and probiotics actively assimilate nitrogen to form the actual macro‐flocs, serving directly as supplementary, immunostimulatory feed for the host. In stark contrast to both of these engineered systems, traditional open ponds driven by diurnal fluctuations, dynamic sediment interactions, and highly resilient native microbiota frequently result in unpredictable colonization and therapeutic outcomes (Hucheng et al. 2020). The implementation of standardized evaluation methods requires a combination of in silico/genomic screening and in vitro antagonism/enzyme profiling and feed processing stability assays and controlled challenges and microbiome metagenomics and multi‐site field trials that examine different water quality factors (Benedetti et al. 2024; Cathers et al. 2022). Synbiotics outperform both probiotics and prebiotics in aquaculture systems yet their effectiveness depends on farm conditions and specific strain selection, which needs on‐farm multi‐site validation for commercial‐scale disease control (Cathers et al. 2022).

### 3.2 Environmental Variables Affecting Efficacy

Environmental parameters act as the primary filters determining the colonization success of administered biologicals. Unlike controlled laboratory conditions, field environments exhibit diurnal and seasonal fluctuations that can drastically reduce probiotic viability.

#### 3.2.1 Temperature Variations

Temperature is the most critical factor influencing metabolic activity and bacterial colonization. The laboratory tests for optimal constant temperatures, but field applications need to handle temperature variations which occur throughout different seasons. Studies conducted in Nile tilapia (*Oreochromis niloticus*) field environments demonstrated that *Lactobacillus plantarum* loses its effectiveness when water temperatures drop below 20°C during winter months. The bacterium enters a dormant state when these conditions occur because it loses its ability to compete against psychrophilic pathogens (Dawood et al. 2020). The high water temperatures which exceed 32°C in tropical shrimp ponds create conditions that make non‐spore‐forming probiotic strains unstable. The system requires heat‐resistant *Bacillus* spores to preserve water quality stability according to Zokaeifar et al. (2012).

#### 3.2.2 Salinity and Osmotic Stress

The main obstacle for probiotic applications in euryhaline species farming stems from changes in salt concentrations. The use of probiotic strains derived from freshwater systems represents a typical mistake which occurs when these strains are applied to brackish or marine environments. Research shows that freshwater lactic acid bacteria experience osmotic shock which prevents them from establishing gut colonization in marine shrimp ( *Litopenaeus vannamei* ). The research conducted a comparative study which showed native marine bacterial strains from local environments provided superior protection to shrimp against *Vibrio* infections than commercial bacterial strains (Castex et al. 2008; Zhang et al. 2021).

#### 3.2.3 pH and Water Chemistry Dynamics

The efficacy of biological additives is also strictly pH‐dependent. The rapid pH fluctuations which happen in high‐intensity biofloc systems because of photosynthesis and respiration cycles make it impossible for beneficial bacteria to execute their enzymatic activities. The research shows *Nitrosomonas* species will halt nitrification when pH levels reach 7.0 or lower, which produces increasing ammonia concentrations regardless of probiotic treatment (Kültz 2022). Furthermore, the presence of organic matter in semi‐intensive earth ponds can sequester probiotic bacteria, reducing their availability to the host. This necessitates higher dosage frequencies in green‐water systems compared to clear‐water RAS environments to achieve the same physiological benefits (Xu and Pan 2013).

### 3.3 Species‐Specific Case Studies

The use of probiotics in intensive tilapia aquaculture systems decreases *Streptococcus agalactiae* mortality because these beneficial microbes fight pathogens through competitive exclusion and produce antimicrobial substances while repairing gut damage and strengthening the immune response. Research conducted in laboratory and field settings demonstrates that *Bacillus* and *Enterococcus* probiotics given through feed or water systems boost survival rates of tilapia (Liu et al. 2021; Suphoronski et al. 2021). The inclusion of prebiotics (FOS/MOS/XOS/GlcOS) at low levels in synbiotic formulations leads to better growth rates and feed conversion ratio (FCR) and water quality improvements which benefits farm economics in pond systems based on reviews and trials with *L. vannamei* and tilapia (Banafsha and Reddy 2025; Hasan and Banerjee 2020). Research shows that three effective control methods for bacterial kidney disease in trout raceways include vaccination and host‐specific probiotics such as *Latilactobacillus* for salmonids and complete filtration systems and biosecurity protocols with hydrocyclones and biofilters (Queiróz et al. 2024). The immunostimulatory and antibacterial properties of medicinal botanicals Moringa and ginger and Vaccinium have proven effective in fin‐fish survival studies which require species‐specific testing in flow‐through systems (Kamble et al. 2024). The most effective method to control early mortality syndrome (EMS) in shrimp involves using protected probiotic feed with micro‐encapsulation or post‐coating and biofloc or synbiotic management and Pseudoalteromonas as an anti‐Vibrio water antagonist (Banafsha and Reddy 2025; Vijayaram et al. 2024).

### 3.4 Economic Impact Assessment

The implementation of biological strategies utilizing probiotics, prebiotics, and synbiotics initially increases feed formulation expenses. The documented economic studies demonstrate that these investments generate returns because the fish survive better and grow stronger from start to finish of their production period (Dias et al. 2020; Widanarni et al. 2020). Research on economic feasibility shows that shrimp and tilapia along with other commercial species achieve better feed utilization efficiency. Organizations can lower their operational costs through reduced feed needs for weight reduction programs which simultaneously protect their financial stability from death‐related losses (Dias et al. 2020; Widanarni et al. 2020). The assessment of biologicals as antibiotic alternatives shows that pharmaceutical drug usage has decreased while environmental pollution from these substances has also decreased. Biological management programs show better long‐term cost‐effectiveness than conventional antibiotic‐based systems according to (Hossain et al. 2024; Nathanailides et al. 2021). The use of biological management protocols for aquatic products allows producers to achieve better market prices for their products. The market advantages exist only under particular regional circumstances and certification systems which require separate market research for each geographic area (Hossain et al. 2024). The system starts generating profits from its initial costs for additive procurement and formulation and staff training because it produces better Feed Conversion Ratios (Das et al. 2022; Widanarni et al. 2020). The field trial results show that these additives behave differently when they encounter different environmental and operational conditions which affect their operational performance. The economic benefits will only become achievable through a complete evaluation of site‐specific elements which Figure 2 demonstrates.

**FIGURE 2:**
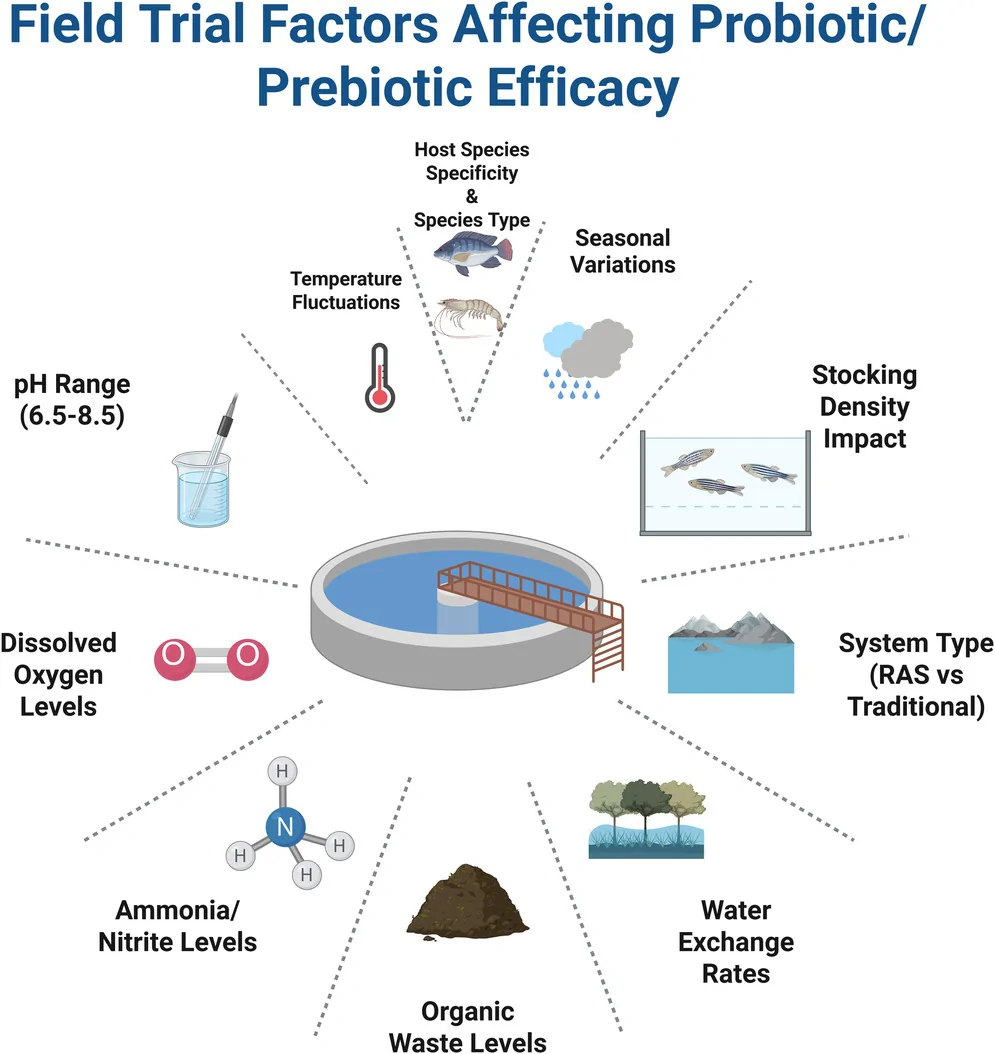
Critical biological, operational, and environmental factors influencing probiotic and prebiotic efficacy in aquaculture field trials. Multiple interconnected variables determine biological control success: Host Species Specificity dictates the anatomical gut architecture, metabolic requirements, and target mucosal surface characteristics unique to each cultured clade, serving as the primary biological filter for allochthonous strain engraftment; pH Range (6.5–8.5) influences probiotic survival rates and prebiotic fermentation processes; Temperature Fluctuations create major changes in microbial functions through Q10 effects and enzymatic activities; Seasonal Variations require dose adjustments and formulation validation for consistent pathogen control; Stocking Density Impact affects organic waste accumulation and pathogen transmission pressure; System Type (RAS vs. Traditional) determines microbiota stability with RAS/biofloc providing controlled conditions while traditional ponds show unpredictable outcomes; Water Exchange Rates influence retention and efficacy of biological agents; Organic Waste Levels from intensive operations challenge biological control capacity; Ammonia/Nitrite Levels affect water chemistry and microbial community dynamics; Dissolved Oxygen Levels influence pathogen‐host–microbe interactions and overall system performance, requiring site‐specific optimization for successful implementation.

## 4 Synergistic Strategies and Innovation

### 4.1 Combined Approaches: Synbiotics and Phyto‐Synbiotics

The addition of synbiotic formulations to manufactured feeds leads to better results than using probiotics or prebiotics alone when testing growth and specific growth rate (SGR) and feed conversion ratio (FCR) and survival in carp production trials according to Pediococcus‐based synbiotics (Qaddoori et al. 2023), whiteleg shrimp using *Bacillus* sp. NP5 combined with honey (Widanarni et al. 2020). The study demonstrated that zebrafish fed Persian shallot with a commercial synbiotic achieved better results than fish fed Persian shallot alone (Ghafarifarsani et al. 2021). Additionally, plant‐extract plus probiotic pairings that have been shown to reduce pathogen impact or enhance immune markers in relevant trials include the use of Persian shallot with a commercial synbiotic (Ghafarifarsani et al. 2021) and *Bacillus* sp. NP5 paired with honey‐derived prebiotics in *Litopenaeus vannamei* (Widanarni et al. 2020), as well as inulin/oligosaccharide prebiotics combined with *Bacillus* and *Lactobacillus* strains in shrimp (Escobedo‐Fregoso et al. 2021). The implementation of multi‐component products containing purified commercial prebiotics results in higher per‐ton additive expenses but manufacturers aim to reduce costs by using local prebiotic substrates and agricultural byproducts such as pineapple and papaya residues which research demonstrates will produce enough FCR and survival benefits to offset the increased expenses in aquaculture (Huynh et al. 2021; Pimpimol et al. 2020). It is important to note that responses to these formulations remain strain–host–system dependent; hence, in vitro preselection and on‐farm validation are crucial before commercial scale‐up (Bledsoe et al. 2022; Śliżewska and Chlebicz‐Wójcik 2020; Śliżewska et al. 2020). The synergistic interaction between probiotics and prebiotics in aquaculture systems demonstrates superior performance outcomes compared to individual component applications, as systematically illustrated through the integrated mechanisms and benefits framework presented in Figure 3.

**FIGURE 3:**
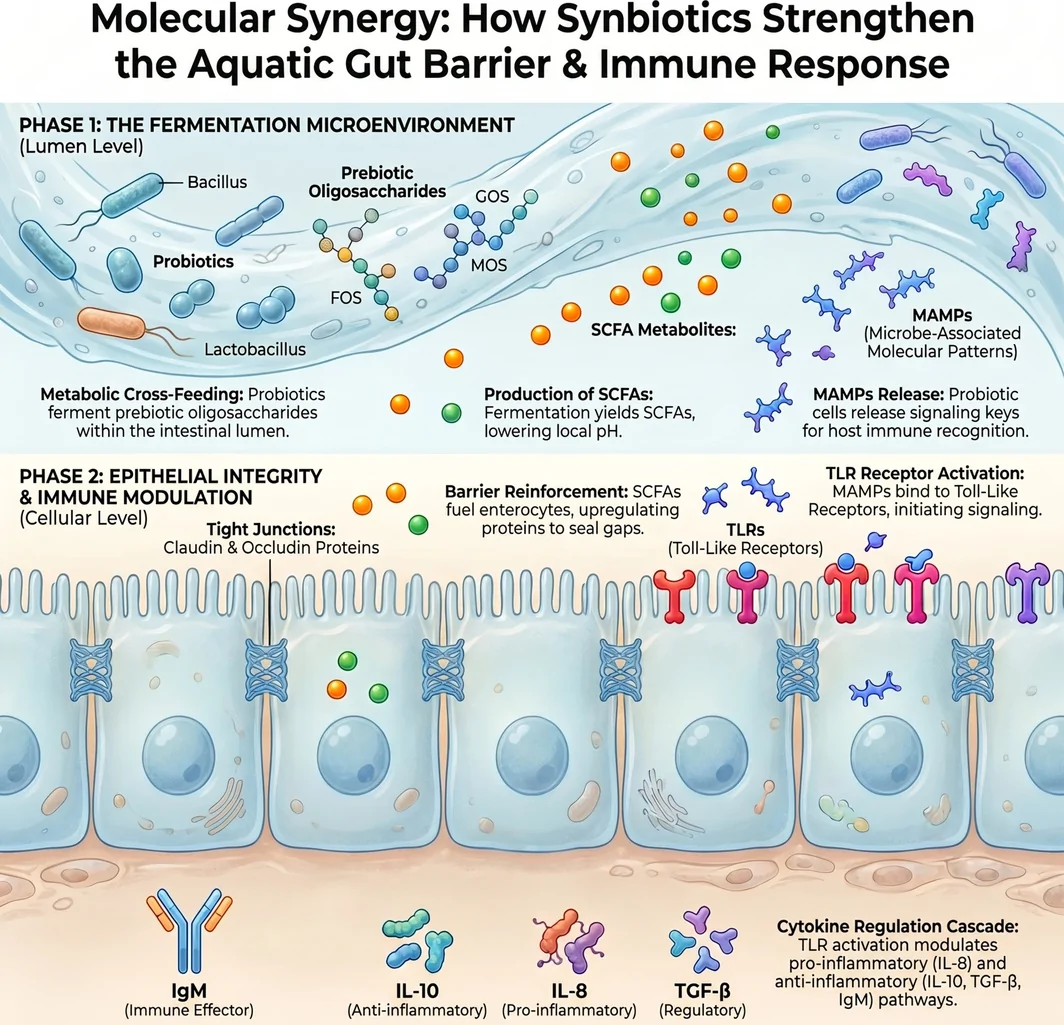
Molecular synergy and multi‐phase pathway dynamics of synbiotics within the aquatic gut barrier and host immune network. Phase 1: The Fermentation Microenvironment (Lumen Level) illustrates metabolic cross‐feeding, wherein administered probiotics (*Bacillus* and *Lactobacillus*) actively ferment dietary prebiotic oligosaccharides—specifically Fructo‐oligosaccharides (FOS), Galacto‐oligosaccharides (GOS), and Mannan‐oligosaccharides (MOS)—within the intestinal lumen. This fermentation process yields short‐chain fatty acid (SCFA) metabolites that lower local luminal pH, while concurrently driving the structural release of Microbe‐Associated Molecular Patterns (MAMPs) essential for host immune recognition. Phase 2: Epithelial Integrity & Immune Modulation (Cellular Level) details host physiological integration. The synthesized SCFAs function as critical oxidative substrates fueling enterocytes, triggering targeted barrier reinforcement by upregulating Claudin and Occludin tight junction proteins to effectively seal intercellular gaps against paracellular pathogen intrusion. Concurrently, shedding MAMPs dock with membrane‐bound Toll‐Like Receptors (TLRs), initiating a robust intracellular signalling cascade. This TLR receptor activation drives a precise Cytokine Regulation Cascade, modulating the expression of pro‐inflammatory factors (IL‐8) and systematically upregulating anti‐inflammatory or regulatory pathways governed by Interleukin‐10 (IL‐10), Transforming Growth Factor‐beta (TGF‐β), and immunoglobulin M (IgM) efferent arms to establish homeostatic tissue resilience.

### 4.2 Physiological Mechanisms of Synergy

The physiological basis of synergy exists because different biological agents activate separate yet matching pathways which strengthen host defences beyond their combined individual effects. The process of metabolic cross‐feeding functions as the main mechanism which enables synbiotic applications to work. The prebiotic component functions as a particular substrate which drives the metabolic operations of the probiotic strain. The fermentation process produces short‐chain fatty acids (SCFAs) at a faster rate which includes butyrate and acetate. The metabolites create an intestinal space with acidic conditions which blocks pathogenic bacteria from multiplying, and they serve as direct energy for enterocytes to enhance gut barrier strength (Ringø et al. 2020; Zeng et al. 2023).

The combination of probiotics with medicinal plants (phytobiotics) results in a process known as facilitated exclusion. Phenols and essential oils in phytochemicals damage Gram‐negative bacterial cell walls and prevent these bacteria from using Quorum Quenching to communicate with each other. The reduction of pathogenic microorganisms creates conditions which allow beneficial probiotic bacteria such as *Lactobacillus* and *Bacillus* to colonize mucosal surfaces while they combat the invading pathogens (Jossefa et al. 2024; Vijayaram et al. 2024). This dual approach exerts precise control over the host immune system through complex, receptor‐mediated molecular pathways rather than merely providing additive benefits. Mechanistically, Microbe‐Associated Molecular Patterns (MAMPs) from probiotics, alongside active phytogenic compounds, are recognized by Pattern Recognition Receptors (PRRs) such as Toll‐like receptors (TLRs) on the epithelial cells of the gut‐associated lymphoid tissue (GALT). This interaction initiates an intracellular signalling cascade that modulates the expression of pro‐ and anti‐inflammatory cytokines and directly stimulates local immune cell proliferation. Crucially, this immune activation is not confined to the intestine. Through the common mucosal immune network, specifically the gut‐skin and gut‐gill mucosal axes, localized GALT stimulation drives the systemic trafficking of primed leukocytes to distal mucosal sites. This systemic cascade results in documented improvements in systemic antioxidant defences, increased secretion of mucosal immunoglobulins (e.g., sIgA/IgT), and elevated lysozyme activity in the skin and gills (Banafsha and Reddy 2025; Latif et al. 2020; Nurzhanova et al. 2021). Consequently, field validation confirms that these mechanistically driven synergistic effects translate into measurable improvements in Feed Conversion Ratio (FCR) and robust resistance against multifactorial mucosal pathogens, although specific strain‐plant combinations require strict molecular optimization tailored to local pathogen profiles (Gede et al. 2024; Ojha et al. 2020; Zhang et al. 2021).

### 4.3 Technological Delivery Systems

The commercial aquafeed industry can use spray‐drying with maltodextrin and gum Arabic and whey matrices and ionic gelation/extrusion to create calcium‐alginate beads and co‐extruded core‐shell structures and hot‐air drying and biopolymer emulsions as practical and affordable encapsulation solutions. The methods have proven their ability to scale up for use in food and biologic supply chain operations (Bennacef et al. 2023; Rahmani‐Manglano et al. 2020; Selim et al. 2021). The protection and controlled release of drugs can be improved through advanced methods including electrospinning and electrospraying and layer‐by‐layer techniques and liposomes/nanocarriers but these methods increase both price and operational complexity (Aguirre‐Güitrón et al. 2022; de Alteriis et al. 2021; Løvschall et al. 2024; Tsekova et al. 2024). The encapsulation process protects the activity during standard 3–6 month storage through its protective barrier system which blocks oxygen and moisture and UV light and decreases water activity and extends germination time and enables controlled substance delivery. The combination of optimized wall materials with drying techniques leads to better enzymatic activity preservation and cell survival rates than free cell methods according to research findings (Barbosa et al. 2022; Machado et al. 2023; Maruyama et al. 2020). The literature indicates that survival of the pelleting process requires two approaches: reducing thermal and steam contact or using heat‐resistant matrices and applying microcapsules as a protective top‐coating. The commercial market selects between spray‐dry and alginate systems for commodity feeds because of their cost‐effectiveness and regulatory compliance and sensory characteristics but uses advanced carriers for premium products (Oliveira Filho et al. 2023; Rahmani‐Manglano et al. 2020; Saberi‐Riseh et al. 2021; Vijayaram et al. 2024).

### 4.4 Multi‐Stress Condition Applications

The maintenance of biological control effectiveness depends on using predictive microbiome and nutritional strategies combined with strict animal care practices and environmental management during seasonal changes and high population densities and transport operations (Churilov et al. 2025; Ciji and Akhtar 2021). The administration of prophylactic timed probiotics and dietary immunostimulants serves as protective measures to prevent immune system suppression caused by stress and subsequent disease susceptibility. Biofloc technology together with water quality management practices help reduce the occurrence of opportunistic pathogens in densely populated aquaculture systems (Hwihy et al. 2021; Marlida 2020). The implementation of adaptive outbreak protocols requires surveillance systems to detect thresholds which activate diagnostic tests for co‐infections and trend analysis to initiate segregation measures and enhanced biosecurity protocols and specific therapeutic interventions and environmental density control (Bass et al. 2023; Sørensen et al. 2024). The effectiveness of multi‐component interventions depends on specific environmental conditions including host factors and co‐infections and thermal and crowding stress levels which need farm‐level molecular and health assessments for validation. The standard programs use pathogen load qPCR tests together with environmental and welfare metrics and transcriptomic and biomarker surveillance to monitor predefined action thresholds which guide adjustments to dosing schedules and husbandry practices (Ellison et al. 2020; Islam et al. 2024; Sørensen et al. 2024; Wang et al. 2021). The success of synbiotic applications in aquaculture depends on the complex interaction of multiple factors, from environmental stressors to pathogen dynamics, requiring comprehensive management strategies as systematically illustrated in Figure 4. Furthermore, building upon these management strategies to ensure long‐term sustainability and resilience against multi‐stress conditions requires a holistic integration of predictive artificial intelligence, multi‐omics frameworks, microalgal biorefineries, targeted phytogenics to mitigate toxicological stressors, and emerging cellular aquaculture technologies (Çelik et al. 2024; Gümüş et al. 2026; Öz 2025; Öz and Üstüner 2026a, 2026b, 2026c, 2026d; Öz, Üstüner, and Çifci 2026; Öz, Üstüner, and Dikel 2026).

**FIGURE 4:**
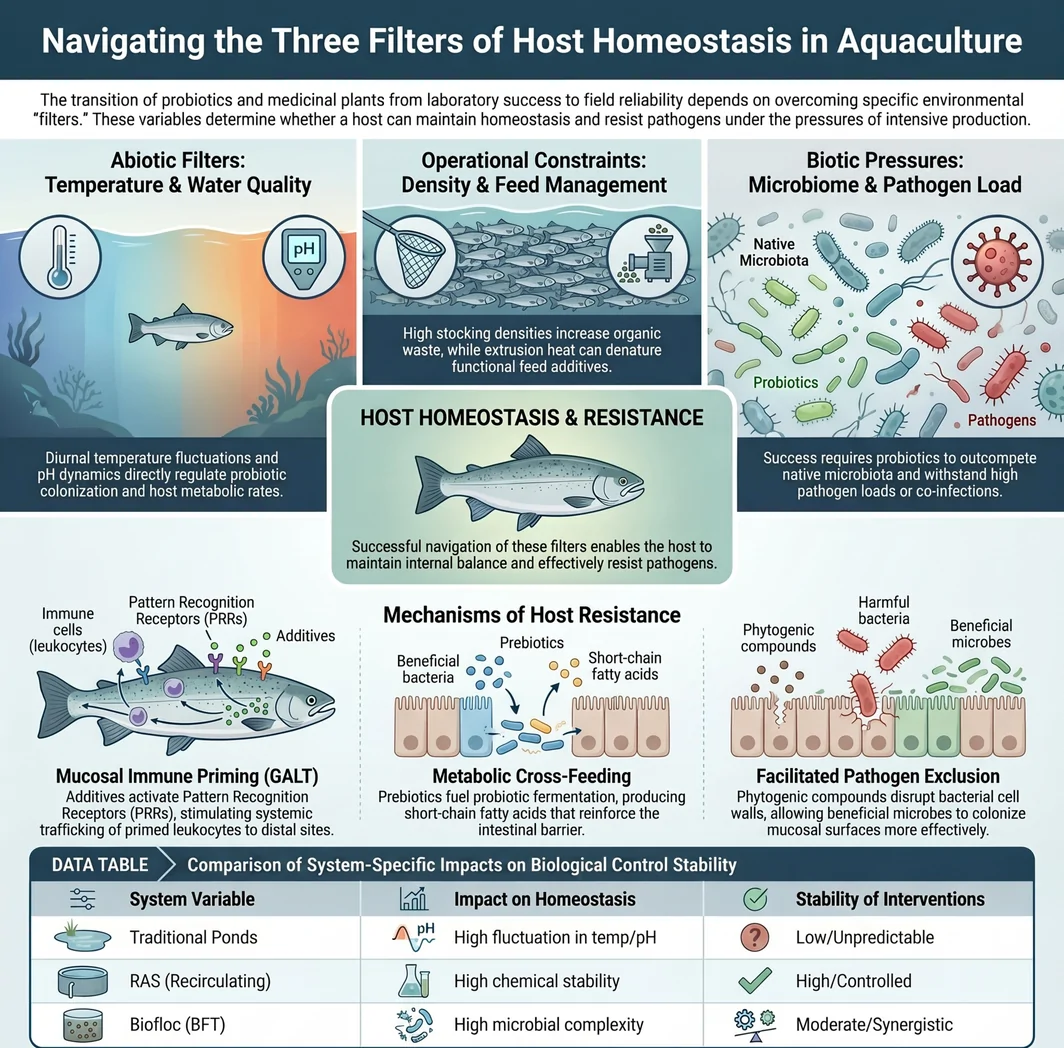
Systems‐aquaculture framework elucidating the hierarchical filters governing host homeostasis, intervention stability, and biological control reproducibility. The schema maps out three key selective barriers determining field success: Abiotic Filters (where diurnal temperature fluctuations and pH dynamics directly modulate probiotic colonization rates and host baseline metabolic efficiency); Operational Constraints (wherein high stocking densities accelerate organic waste accumulation, and thermal processing profiles during feed extrusion risk denaturing active functional components); and Biotic Pressures (which demand that administered bio‐additives successfully outcompete the resilient native microbiota while withstanding high pathogenic bio‐burdens or complex polymicrobial co‐infections). Successful navigation of these overlapping filters enables the preservation of internal Host Homeostasis & Resistance. The underlying mechanisms of host resistance are driven by three coordinated axes: Mucosal Immune Priming via Gut‐Associated Lymphoid Tissue (GALT), where functional additives activate specific Pattern Recognition Receptors (PRRs) to stimulate systemic leukocyte trafficking to distal mucosal sites; Metabolic Cross‐Feeding, which fuels SCFA generation to fortify intestinal mechanical barriers; and Facilitated Pathogen Exclusion, where phytogenic botanical derivatives disrupt target bacterial cell walls to promote competitive surface colonization by beneficial microbes. The integrated data matrix evaluates these variables across specific culture configurations, showing that while traditional ponds introduce high temperature/pH fluctuations leading to unpredictable intervention stability, Recirculating Aquaculture Systems (RAS) secure high chemical stability and controlled reproducibility, and Biofloc Technology (BFT) presents high microbial complexity that fosters moderate, highly synergistic biological control dynamics.

## 5 Challenges and Implementation

### 5.1 Product Quality, Stability, and Regulatory Issues

Fish farming with probiotics needs solutions which solve three main problems that stem from operational difficulties and product instability and fish host requirements and regulatory compliance standards (Ishthiaq et al. 2021). The viability of probiotics faces negative impacts from environmental factors which include temperature and humidity and light exposure and oxygen sensitivity (Fachri et al. 2024; Sun et al. 2023). The survival rate and metabolic activity of probiotics becomes less effective when the environment temperature increases. Therefore, storage conditions and special formulations are of great importance in aquaculture (Aguinaga Bósquez et al. 2022). The implementation of encapsulation techniques for stability maintenance protects probiotics from environmental elements which occur during manufacturing and storage and gastrointestinal transit so they remain active until their required time (Sun et al. 2023). The research results from this study confirmed the findings of Valle Vargas et al. (2025), who found that Nile tilapia benefited more from probiotic encapsulation than from using non‐encapsulated probiotics. The *S. cerevisiae* strain survived feed processing at high temperatures when researchers from Bevilacqua et al. (2020). Probiotics have been encapsulated using different techniques, and since each technique has its own disadvantages, it is necessary to overcome these difficulties to ensure that probiotics remain viable and have a long shelf life.

To accurately contextualize the technological necessity of these delivery systems, it is essential to establish a viability risk scale comparing different aquaculture categories and host feeding ecologies. From an operational system perspective, traditional open ponds represent the highest risk to microbial viability due to intense exposure to uncontrolled abiotic fluctuations (e.g., UV radiation, temperature spikes). Recirculating Aquaculture Systems (RAS) pose a moderate risk mitigated by chemical buffering, whereas Biofloc Technology (BFT) presents the lowest viability risk since functional microbes are applied directly as water additives, entirely evading feed processing trauma. Furthermore, host feeding habits introduce a critical biological viability risk. Carnivorous species (e.g., salmonids, marine finfish) are subjected to a significantly higher risk profile compared to omnivorous or herbivorous species like carp. The formulation of carnivorous diets requires high‐protein, lipid‐rich matrices that demand extreme thermal and mechanical shear forces during industrial extrusion, causing massive probiotic mortality prior to consumption. Upon ingestion, the carnivorous gastrointestinal tract presents a highly hostile environment characterized by a strictly acidic stomach (low pH) and rapid transit times. In stark contrast, stomachless omnivores (cyprinids) utilize standard pelleted feeds manufactured at lower temperatures and possess longer, pH‐neutral intestinal tracts, naturally facilitating superior probiotic engraftment. Therefore, advanced delivery strategies like microencapsulation are not universally required; they are strictly mandatory for high‐risk carnivorous diets but may be optional for low‐risk systems and species.

The quality control process for probiotics requires Whole Genome Sequencing (WGS) because this method enables scientists to analyse genes at their most advanced level (Wang et al. 2020; Mustafa 2024). WGS technology enables us to select useful bacterial strains which possess immune system regulation abilities and enzyme production and pathogen fighting capabilities, but it also reveals their potential antibiotic resistance traits (Wang et al. 2020; Raj et al. 2023).

The selection of suitable probiotic strains stands as the main challenge which scientists must solve. The right strain selection plays a major role in the effectiveness of probiotics in terms of their resistance to environmental conditions (Wendel 2022; Chizhayeva et al. 2022). The multiplication of probiotics in water depends on their reproduction speed being higher than their shedding rate because this requires appropriate strain selection (Fachri et al. 2024). The host specificity of probiotics functions as a restrictive element because different species need distinct probiotic strains to achieve therapeutic success (Ordanel et al. 2025). The identification of specific probiotic formulations which correspond to various species becomes necessary to enhance probiotic effectiveness in aquaculture operations. The method generates a new challenge because scientists must create particular probiotic solutions which correspond to the bacterial populations of different fish species (Das et al. 2022; Ordanel et al. 2025). The identification of these differences becomes possible through individual fish tracking methods and extensive research (Lowe et al. 2020).

The different regulatory systems which control probiotic usage across different geographic areas create obstacles for their implementation (Lulijwa et al. 2020). The solution to these obstacles demands organizations to collaborate with regulatory bodies while understanding current laws and conducting scientific research to validate probiotic effectiveness (Hancz 2022). The European Union needs probiotics to meet European Food Safety Authority (EFSA) established criteria which determine their suitability for aquaculture use (Koutsoumanis et al. 2021). The requirements demand researchers to conduct extensive strain‐by‐strain investigations while they must evaluate antibiotic resistance gene transmission which leads to longer development periods and higher expenses (Zavišić et al. 2023).

### 5.2 Dosing, Duration, and Farm Logistics

In aquaculture, the frequency and duration of application, dosage, and feeding strategies for medicinal plant products and probiotics used as protective agents against diseases are of great importance in achieving the desired yield. For dosage to be effective, the LD50/LC50 value of the pathogen in the target organism must be known (Van Doan et al. 2021). LD50 (lethal dose 50%) refers to the dose that causes death in 50% of the individuals tested. LC50 (lethal concentration 50%) refers to the concentration that is lethal to 50% of the individuals tested.

The application techniques for this product include three different methods which include feed mixing, water soaking, and injection procedures. Each technique can produce different responses depending on the interaction conditions (Chizhayeva et al. 2022). The application techniques need optimization through consideration of water quality together with fish behaviour and feed conversion efficiency (Fachri et al. 2024). The species needs specific applications which duplicate their native environment and their specific dietary needs to reach their highest potential (Wuertz et al. 2021). For example, bath treatment is particularly effective in the early stages of the larval phase. A study conducted with zebrafish larvae found that the treatment increased growth, development, and survival rates (Padeniya et al. 2022). Another study using dietary *Nigella sativa* oil positively affected growth performance, oxidative stress regulation, physiological balance, and tissue architecture in Nile tilapia (Öz, Üstüner, Çifci, et al. 2025; Öz, Üstüner, Jumayeva, and Dikel 2025).

### 5.3 Context‐Dependency and Reproducibility—Farm‐to‐Farm Variability

Recently, prebiotics, probiotics, and medicinal herbal products have been used in aquaculture to enhance growth, accelerate development, and support the immune system. When used as a feed additive, probiotics appear as a component that supports the health of the host. Prebiotics, on the other hand, are known as compounds that stimulate the growth of a specific number of microbes. However, these products face certain problems in field applications. Context‐dependency and reproducibility issues are often prominent. Many environmental factors, such as microbial changes and climate changes, that naturally occur in open water aquaculture systems are not observed in closed aquaculture systems. In closed systems, culture proceeds under specific standards and control. However, in closed systems, many products are used to maintain the chemical balance of the water. This can alter the bioload and microbiota over time. While prebiotics, probiotics, and medicinal herbs used in these two systems are generally used for fish development and health, the benefits of this use vary from farm to farm and system to system (Fossmark et al. 2020; Lorgen‐Ritchie et al. 2023).

In aquaculture, context dependence is used to describe different outcomes. However, this definition may be insufficient for clarity. It can be defined as the effects and consequences of prebiotics, probiotics, or medicinal plants used in aquaculture differ depending on many parameters, such as the fish species, environmental factors, the type of probiotic strain administered, the dosage applied in the diet, the host's antibiotic history, the host's age, diet, and disease history (Amenyogbe et al. 2024; Catford et al. 2022). While aquaculture products are thought to have a specific gut microbiota under normal conditions, this can vary depending on factors such as the salinity of the living environment, the oxygen level of the water, changes in feeding habits, stress levels, climatic conditions, and stocking density. Furthermore, this pattern can vary from farm to farm (Amenyogbe et al. 2024). For example, considering stocking density in different culture areas, a decrease in digestive enzyme activity due to stress will occur in a crowded environment (Dong et al. 2018). In a different example, abalone ( *Haliotis laevigata* ) shows a decrease in trypsin and amylase activity with age (Bansemer et al. 2016). In this regard, while prebiotic, probiotic, and medicinal plants clearly show benefits in one cultivation system, they may not provide as much benefit as expected in another (Amenyogbe et al. 2024).

Simply defined, reproducibility is the finding that the results are significantly similar when the data obtained from a study are repeated in a different time period, in a different environment, or with different species (Killen et al. 2016; Takács et al. 2016). One of the most significant problems encountered in terms of reproducibility in aquaculture is farm‐to‐farm variability. The effects of a therapeutic agent vary depending on variables such as the genetics of the aquatic products, the structure of the aquatic plants, the composition of the feed and the method of application, and the microbiota of the region. It is known that the effects of the applied prebiotics and probiotics vary depending on the dominant role of the gut microbiota of the organism. In this case, the microbiota of the region should not suppress or disrupt the composition of the intended agent. Otherwise, the active agent may not establish the desired establishment in the gut microbiota and may affect the results of the study (Calcagnile et al. 2024). For example, in a study conducted by Srisapoome and Areechon (2017), live *Bacillus pumilus* was obtained from Nile tilapia ( *Oreochromis niloticus* ) farms. The collected *B. pumilus* was tested on both laboratory and farm animals. In this experiment, conducted with the same feed concentration, the phagocytic activity and superoxide anion levels of the laboratory animals indicated that immunity was supported and they also showed resistance against *Streptococcus agalactiae* . When this was examined in farm animals, resistance to *S. agalactiae* was reported only in early and mid‐April, when the air temperature reached 33°C. In another study, Nile tilapia ( *Oreochromis niloticus* ) were divided into two groups. Group A (71.4 ± 4.4 g) was fed a commercial feed coated with *Bacillus velenzensis* and BiOWiSH Feedbuilder Syn3. Group B (5.34 ± 0.42 g) was fed a diet coated with BiOWiSH Feedbuilder Syn3. The trial results showed no difference in growth performance, but improvements in water quality and microbiota were observed. It was emphasized that results may vary depending on the probiotic concentration and route of administration (Oliveira et al. 2025).

### 5.4 Ethical, Environmental, and Biosafety Considerations—Ecosystem Impacts, AMR Perspective

Today, consumers have begun to rely heavily on aquaculture to meet their protein needs. This has led to the proliferation and increasing importance of aquaculture farms. To meet this growing demand, both open and closed aquaculture systems have been established. While stocking density varies in these systems, a number of measures are implemented to protect this density against disease. Supplements such as medicinal plants, antibiotics, feed additives, vitamins, prebiotics, and probiotics are used to maintain the health of the cultivated crops (Lorgen‐Ritchie et al. 2023; Amenyogbe et al. 2020). Live and inanimate feed additives such as prebiotics, probiotics, and medicinal plants have been researched for years to improve human and animal health or strengthen against harmful microorganisms (Todorov et al. 2024). Probiotics increase the host's resistance to diseases and also increase the host's tolerance to all possible stressors. Probiotics and prebiotics used in both in vitro and in vivo experiments have been reported to strengthen immunity and promote growth (Calcagnile et al. 2024). However, this can sometimes lead to negative consequences. The probiotic bacteria used can migrate into the water and overwhelm many bacterial species in the environment, disrupting the microbial balance of the habitat. Furthermore, aquaculture products normally present in the region may be indirectly negatively affected by the presence of a microbiota foreign to the feed ingredients used in commercial farming. The use of some medicinal plants in large quantities or for extended periods not only has negative effects on living organisms but can also affect the microbial diversity of the region (Reverter et al. 2014; Grigorakis 2010). To prevent these adverse events, attention must be paid to various practices. First, factors such as temperature, humidity, and oxygen affect the viability of probiotic bacteria, prebiotics, and medicinal plants. Therefore, appropriate storage and field use conditions must be ensured. Furthermore, the host specificity and dosage of probiotics should be considered. A particular strain may benefit one fish species but not another. Therefore, it would be more beneficial to choose broad‐spectrum strains and determine and apply the appropriate dose. When necessary, non‐viable forms should be preferred. Additionally, environmental risks should be considered, and the resulting market release should be made. Furthermore, microbial and metagenomic analyses of both the in‐water population and the direct water should be conducted, and an appropriate strain and dosage should be applied accordingly (Fachri et al. 2024).

From an ethical perspective, numerous studies have been conducted using probiotics, prebiotics, and medicinal plants for the welfare and health of aquaculture. These studies not only address the health and welfare of aquaculture but also directly impact human nutrition and health. Furthermore, labour and feed costs, as well as all other expenses, are impacted throughout the entire process from production to consumption. Using these products at inappropriate doses or in incorrect formulations has been reported to cause problems such as reduced digestibility, decreased extracellular enzyme production, decreased growth rate, high feed conversion ratio (FCR), and decreased immune response (Hamdan et al. 2016; Makled et al. 2019; De Marco et al. 2023). For example, in one study, Nile tilapia fed a 10% probiotic diet had higher FCR test results than those fed lower probiotic doses, emphasizing that 10% probiotics was an overdose for tilapia (Khunrang et al. 2021).

Antibacterial drugs are used to prevent and treat bacterial diseases in aquaculture. Unnecessary, prolonged, or high‐dose use of these drugs leads to the development of resistance in zoonotic bacteria. These resistant bacteria are transmitted to humans through the consumption of cultivated products. Aquatic environments provide the necessary environment for the development of antimicrobial resistance (AMR) in such bacteria (Milijasevic et al. 2024). AMR occurs in two forms: natural resistance and acquired resistance. Natural resistance is transmitted from the parent cell to the offspring and can persist in its existing resistance to antibiotic use. However, this is not the case with acquired resistance. Acquired resistance arises as a result of exposure to antimicrobial agents. In bacteria, it can occur through DNA mutation, horizontal gene transfer (HGT), or the acquisition of resistance genes from other bacteria. In the aquaculture field, there are many ways for AMR to be transmitted from one bacterium to another. For example, a foreign bacterium introduced into water via HGT can rapidly spread its genes within the surrounding population. Considering this example from the perspective of probiotic bacteria, many commercial probiotic bacteria are used in the aquaculture feed industry. As a result of the presence of AMR in the probiotic bacteria used in these feed compositions, AMRs are easily transmitted to the surrounding bacteria via HGT. This harms the microbiota (Milijasevic et al. 2024; Yuan et al. 2023). The consequences of all these events raise biosecurity concerns (Milijasevic et al. 2024).

## 6 The Road Ahead: Emerging Directions in Disease Management

### 6.1 Precision Monitoring for Biological Stability

Digital health applications represent a paradigm shift from reactive treatment to predictive, mechanistic management in precision aquaculture (O'Donncha and Grant 2019). Because the success of biological interventions is strictly dependent on environmental stability, the implementation of sensors and Internet of Things (IoT) connectivity is crucial for the real‐time tracking of abiotic parameters, helping to maintain exact conditions that support probiotic survival (Liu et al. 2025). Going beyond basic monitoring, digital twin technology assimilates real‐time sensor data to create highly accurate virtual representations of physical entities. This allows for the dynamic control of closed systems by mathematically simulating how organisms will react to external environmental fluctuations before physical application (Føre et al. 2024). Ultimately, artificial intelligence models process these multifactorial datasets to identify early pathogen emergence and calculate the precise temporal windows for biological additive application, preventing field‐level failures (Öz, Üstüner, Çifci, et al. 2025; Öz, Üstüner, Jumayeva, and Dikel 2025).

### 6.2 Next‐Generation Microbiome Interventions

The teleost gut microbiota is not merely a passive bystander but an active orchestrator of host health, executing essential functions such as mucosal barrier maintenance, energy metabolism regulation, and endocrine‐immune system influence (Luan et al. 2023). Consequently, managing this microbiome has transitioned from an observational concept to a highly targeted strategy for sustainable aquaculture production (Ruiz, Gisbert, and Andree 2024; Wang et al. 2018). To overcome the viability losses of live probiotics during commercial processing, research currently investigates next‐generation combinations involving phage‐probiotics and postbiotics to secure disease resistance, animal growth, and feed efficiency (Ruiz, Torrecillas, et al. 2024). Postbiotics—inactivated cells and their metabolites—deliver exact doses of signalling molecules that enhance gut bacterial development and prime the immune system without the viability constraints of live cells (Yao Ang et al. 2020). For example, postbiotics derived from *Aeromonas sobria* , *Weissella cibiaria*, and *Bacillus subtilis* have demonstrated profound immunomodulatory defences against *Yersinia ruckeri* in rainbow trout (Quintanilla‐Pineda et al. 2024), while *Vibrio proteolyticus* postbiotics significantly enhance sea bream microbiota and systemic immune functions (Pérez‐Gómez et al. 2025). Concurrently, phage therapy provides a critical precision tool; by utilizing commercially available products for specific pathogen elimination, producers can effectively clear mucosal niches, thereby protecting and facilitating the colonization of beneficial probiotic microorganisms (Rai et al. 2024).

### 6.3 Omics Validation of Functional Additives

To transition from descriptive phenotypic observations to concrete molecular validation, the integration of multi‐omics technologies is indispensable. This multi‐layered approach simultaneously characterizes the genome, transcriptome, proteome, and metabolome, allowing bioinformatic systems to precisely map the host's complex pathway responses to specific prebiotic or probiotic stimulation (Li et al. 2020; Natnan et al. 2021). Metabolomic technology, for instance, uncovers the exact biochemical mechanisms and signalling molecules involved in manifesting immunological effects and defending against pathogen threats (Low et al. 2017; Natnan et al. 2021). Genomic studies identify expression patterns and genetic locations, pinpointing the specific molecular pathways responsible for physiological traits and behavioural changes (Suravajhala et al. 2016), which are critical for breeding genetically robust stocks with enhanced disease resistance (Bernatchez et al. 2017; Yáñez et al. 2019). Proteomics systematically targets the entire proteome to identify subtle cellular conditions under stress, providing essential data across disease management, nutrition assessment, and animal welfare (Almeida et al. 2015; Rodrigues et al. 2012). Concurrently, transcriptomic research elucidates the dynamic regulation of immune‐related genes and specific host immune responses against diseases (Salinas and Magadán 2017), driving the development of new diagnostic methods (Natnan et al. 2021). Ultimately, these omics technologies enable medical professionals to detect diseases early through the discovery of highly sensitive diagnostic biomarkers (Natnan et al. 2021; Trzeciak et al. 2020), with multiple definitive biomarkers already identified across genomic, proteomic, and metabolomic domains to standardize functional additive efficacy (Geng et al. 2015; Liu et al. 2016, 2020).

## 7 Conclusion

The transition of probiotics and prebiotics and medicinal plants from laboratory promise to commercial reliability is currently hindered by environmental inconsistencies and formulation challenges. While in vitro studies consistently demonstrate pathogen inhibition, field efficacy is frequently compromised by variables specific to the production system such as fluctuating water chemistry and competitive exclusion by native microbiota and the harsh conditions of feed manufacturing. This review highlights that the implementation gap is largely driven by a mismatch between the metabolic requirements of biological strains and the dynamic realities of open production systems.

Future strategies must move beyond generic approaches to bridge this gap. The integration of precision aquaculture technologies including water quality monitoring using the Internet of Things and dosing protocols guided by the microbiome offers a pathway to synchronize biological interventions with the physiological status of the host. Furthermore, the adoption of advanced delivery systems such as microencapsulation is essential to ensure that therapeutic dosages reach the gut in a viable state in intensive biofloc and recirculating aquaculture systems.

Biological additives represent the most viable alternative to antibiotics for ensuring the sustainability of aquaculture over the long term. However, their success depends on a paradigm shift toward precision management where strain selection is ecologically validated and delivery is technologically protected and application is driven by data. Addressing these pillars will transform these biological tools from inconsistent supplements into predictable and essential components of global aquatic food security.

## Author Contributions

**Mustafa Öz:** conceptualization, writing – review and editing, writing – original draft, data curation, supervision, resources, software, validation, visualization. **Suat Dikel:** conceptualization, writing – original draft, writing – review and editing. **Emin İleri:** writing – original draft, writing – review and editing. **Sümmani Çifci:** conceptualization, visualization, writing – review and editing, writing – original draft. **Furkan Budak:** writing – original draft, writing – review and editing. **Enes Üstüner:** conceptualization, writing – original draft, writing – review and editing, validation, software, resources, visualization.

## Funding

The authors have nothing to report.

## Ethics Statement

The authors have nothing to report.

## Consent

The authors have nothing to report.

## Conflicts of Interest

The authors declare no conflicts of interest.

## Data Availability

All data and materials used in this review are drawn from published sources and properly cited in the manuscript. No new datasets were generated or analysed during the current study.
